# Diversity, phylogenetic distribution, and origins of venomous catfishes

**DOI:** 10.1186/1471-2148-9-282

**Published:** 2009-12-04

**Authors:** Jeremy J Wright

**Affiliations:** 1 Department of Ecology and Evolutionary Biology, University of Michigan, Ann Arbor, MI 48109, USA

## Abstract

**Background:**

The study of venomous fishes is in a state of relative infancy when compared to that of other groups of venomous organisms. Catfishes (Order Siluriformes) are a diverse group of bony fishes that have long been known to include venomous taxa, but the extent and phylogenetic distribution of this venomous species diversity has never been documented, while the nature of the venoms themselves also remains poorly understood. In this study, I used histological preparations from over 100 catfish genera, basic biochemical and toxicological analyses of fin spine extracts from several species, and previous systematic studies of catfishes to examine the distribution of venom glands in this group. These results also offer preliminary insights into the evolutionary history of venom glands in the Siluriformes.

**Results:**

Histological examinations of 158 catfish species indicate that approximately 1250-1625+ catfish species should be presumed to be venomous, when viewed in conjunction with several hypotheses of siluriform phylogeny. Maximum parsimony character optimization analyses indicate two to three independent derivations of venom glands within the Siluriformes. A number of putative toxic peptides were identified in the venoms of catfish species from many of the families determined to contain venomous representatives. These peptides elicit a wide array of physiological effects in other fishes, though any one species examined produced no more than three distinct putative toxins in its venom. The molecular weights and effects produced by these putative toxic peptides show strong similarities to previously characterized toxins found in catfish epidermal secretions.

**Conclusion:**

Venom glands have evolved multiple times in catfishes (Order Siluriformes), and venomous catfishes may outnumber the combined diversity of all other venomous vertebrates. The toxic peptides found in catfish venoms may be derived from epidermal secretions that have been demonstrated to accelerate the healing of wounds, rather than defensive crinotoxins.

## Background

The venoms produced by cnidarians, mollusks, snakes, arachnids, insects, and some mammals have been the subject of multiple studies of chemical structure [1-3], pharmacology [2-5], and toxicology [5-7], in addition to several evolutionary studies [8-12], but information regarding these aspects of fish venoms is relatively sparse [13-18]. Until recently, even reliable estimates of the number of venomous fish species have been unavailable. Morphological examinations, combined with phylogenetic analyses have suggested that 585-650 species of spiny-rayed fishes are venomous, a number which rivals the known diversity of venomous snakes and is significantly higher than previous estimates of about 200 venomous spiny-rayed fish species [18]. We still lack estimates, however, for catfishes (Order Siluriformes), a diverse, monophyletic group with 34 recognized extant families and over 400 genera containing more than 3,000 known species [19]. The historical lack of such basic information may be largely responsible for the paucity of research on venomous fishes in general, and venomous catfishes in particular.

**Figure 1:**
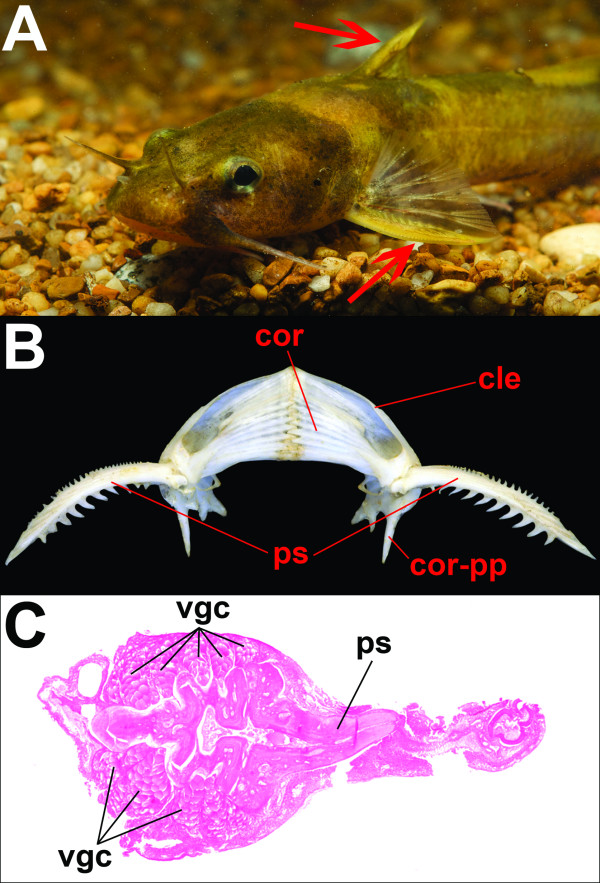
**The Venom Delivery System of Catfishes**. (**A**) Northern madtom (*Noturus stigmosus*) with dorsal and pectoral fin spines indicated by red arrows. (**B**) Pectoral girdle of *Noturus stigmosus *with articulated pectoral fin spines. Abbreviations: ps = pectoral fin spine, cle = cleithrum, cor = coracoid, cor-pp = posterior process of coracoid. (**C**) Cross section of the pectoral-fin spine of *Noturus stigmosus *showing the association of venom gland cells with the fin spine. Abbreviations: ps = pectoral spine, vgc = venom gland cells.

The venom glands of catfishes are found in association with sharp, bony spines along the leading edge of the dorsal and pectoral fins, which can be locked into place when the catfish is threatened (Fig. 1). When a spine enters a potential predator, the integument surrounding the venom gland cells is torn, releasing venom into the wound. Catfish venoms have been shown to display neurotoxic and hemolytic properties and can produce a variety of additional effects such as severe pain, ischemia, muscle spasm, and respiratory distress; though any single species' venom may not display all of these properties [20]. These effects are produced in a wide range of taxonomic classes of vertebrates, including mammals, reptiles, birds, and amphibians [21]. In humans, the primary symptoms are severe pain and swelling at the site of envenomation, though fatalities have been reported in cases involving *Plotosus lineatus *(Plotosidae) and *Heteropneustes fossilis *(Clariidae) [20]. Complications arising from secondary infection of the wound are also frequently encountered.

The chemical nature of piscine venoms is poorly known, though the loss of toxicity seen when these venoms are subjected to common denaturing agents suggests that proteins constitute the major toxic component of these secretions [16]. Thus far, detailed examinations of these proteins in catfishes have been limited to the venoms of *Plotosus canius*, a particularly toxic marine species found in Southeast Asia, and *Ameiurus catus*, a freshwater species found in the eastern United States. The neurotoxic and hemolytic properties of *P. canius *venom have been attributed solely to a 15 kDa protein, termed toxin-PC [22]. The venom of *A*. *catus *was thought to contain anywhere from two to eight toxic proteins with approximate molecular weights of 10 kDa [23]. Both the mechanism by which these toxins act and their physiological targets are very poorly understood. It is thought that cytolytic activity due to pore formation in cell membranes is a likely explanation, as this activity is present in other 'pain-producing' venoms, such as those produced by bees [24] and platypus [25], and reactions consistent with this mechanism have been observed in response to piscine venoms [16].

As a globally distributed and thus, biogeographically interesting group, catfishes have recently been a topic of interest in several phylogenetic studies [26-29]. When combined with these data, information regarding the distribution of venom glands within the Siluriformes can be examined in an evolutionary context, and we can begin to build a foundation to advance the studies of venom evolution in this group to the level seen in other venomous organisms. In this work, I use histological and toxicological techniques to elucidate the diversity and taxonomic distribution of venomous catfishes and examine these findings within the phylogenetic framework established by previous authors to provide a broad-scale hypothesis for the evolutionary origins of venom glands in catfishes. These examinations are further integrated with preliminary biochemical characterizations of venoms from several catfish species to highlight an intriguing, novel hypothesis for the evolutionary development of venom glands in catfishes.

## Results

**Figure 2:**
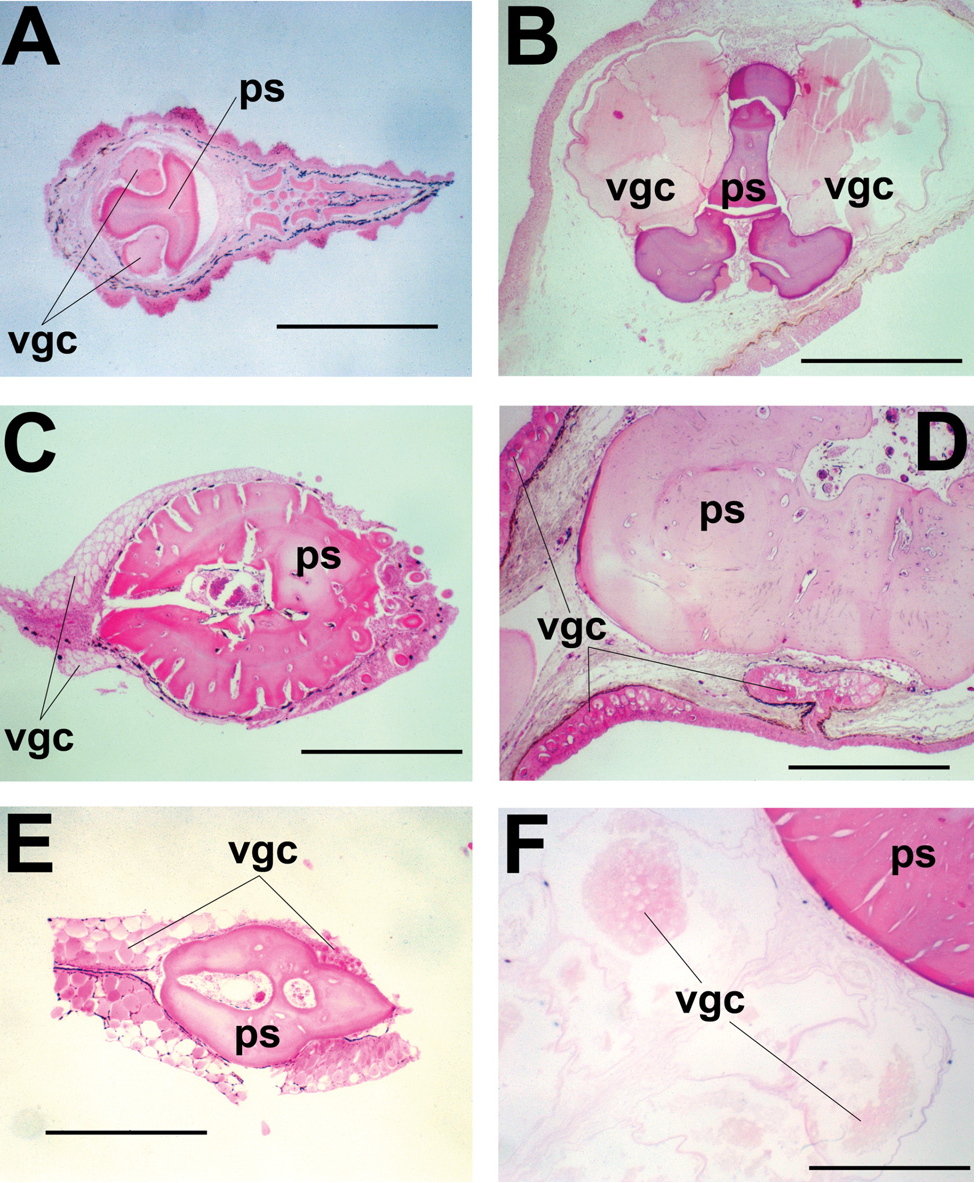
**Histological preparations of fin spines from several venomous catfish species**. (**A**) *Acrochordonichthys rugosus *(Akysidae), (**B**) *Liobagrus reini *(Amblycipitidae), (**C**) *Dianema longibarbis *(Callichthyidae), (**D**) *Chaca chaca *(Chacidae), (**E**) *Lophiobagrus cyclurus *(Claroteidae), (**F**) *Lithodoras dorsalis *(Doradidae). Abbreviations: ps = pectoral fin spine, vgc = venom gland cells. Scale bars, 0.5 mm.

**Figure 3:**
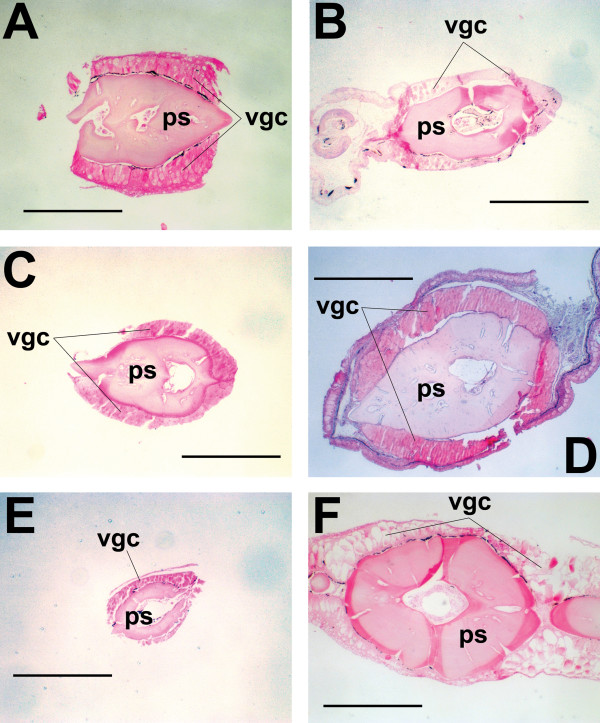
**Additional histological preparations of fin spines from venomous catfish species**. (**A**) *Pimelodella mucosa *(Heptapteridae), (**B**) *Chiloglanis productus *(Mochokidae), (**C**) *Pseudolais pleurotaenia *(Pangasiidae), (**D**) *Plotosus canius *(Plotosidae), (**E**) *Schilbe mystus *(Schilbidae), (**F**) *Horabagrus brachysoma *(*incertae sedis*). Abbreviations: ps = pectoral fin spine, vgc = venom gland cells. Scale bars, 0.5 mm.

To establish a preliminary estimate of the number and phylogenetic distribution of venomous catfish species, 159 species from over 100 genera, representing 32 of the 34 siluriform families were examined for the presence of venom glands (Additional file 1). Material for representatives of the families Austroglanididae and Lacantuniidae was unavailable for study, but their omission from this study has little effect on estimates of the number of venomous catfish species, due to the low species diversity of these families (three species and one species, respectively). Structures identified as venom glands were observed in 20 families. Venom gland size, orientation, and cellular morphology were found to vary considerably between, and sometimes within, families (Additional file 1; Figs. 2, 3). Based upon the generic identity of the venomous species identified, the number of species contained within those genera, and the number of remaining unexamined species in those families shown to contain venomous representatives (See Methods for detailed explanation), an estimate of 1234-1625 venomous catfish species was developed (Table 1).

**Table 1:** Taxonomic distributions and estimates of venomous catfish diversity.

Taxon	# Presumed Venomous
Siluriformes - Catfishes	≈1250-1625 species
Akysidae - Asian stream catfishes	48
Amblycipitidae - Torrent catfishes	26-28
Anchariidae - Madagascan catfishes	4-6
Ariidae - Sea catfishes	67-134
Bagridae - Bagrid catfishes	176-198
Callichthyidae - Armored catfishes	182-194
Chacidae - Angler catfishes	3
Clariidae - Labyrinth catfishes	79-114
Claroteidae - Claroteid catfishes	56-84
Cranoglanididae - Armorhead catfishes	3
Doradidae - Thorny catfishes	48-81
Heptapteridae - Shrimp catfishes	91-160
Ictaluridae - North American catfishes	57-64
Mochokidae - Squeakers	166-189
Pangasiidae - Shark catfishes	27-30
Pimelodidae - Antennae catfishes	41-79
Plotosidae - Eeltail catfishes	17-37
Pseudopimelodidae - Bumblebee catfishes	21-31
Schilbidae - Glass catfishes	48-62
Siluridae - Sheat catfishes	74-83

Basic estimates of family diversity used to generate these figures are taken from [19], and were supplemented through consultation of species descriptions that have been published since the completion of that study.

The production of toxic compounds by representatives from several siluriform families was confirmed through analysis of effects of crude fin-spine extracts on a predatory fish species. The injection of fin-spine extracts caused symptoms of envenomation in all cases; in all cases but one (*Plotosus lineatus*), injection with control extracts prepared from fin tissue yielded no appreciable effect. Symptoms produced by the venoms tested included chromatophore expansion at the injection site, loss of coloration elsewhere on the body, hemorrhage, loss of equilibrium, muscle spasm, and in one instance (*Plotosus lineatus*), rapid mortality (Table 2). Symptoms of envenomation occurred immediately and were resolved within an hour in most trials. Though representatives from several families were not examined, species in those families possess cells associated with their fin spines that have similar, if not identical, morphologies to the venomous species tested, suggesting that these cells produce toxic substances in the untested families as well.

**Table 2:** The effects of several catfish species' venoms on Largemouth Bass.

	**Venom Effect**					
**Species**	**Color loss**	**Myoclonus**	**Tetanus**	**Hemorrhage**	**Loss of Equilibrium**	**Mortality**
*Arius jordani *(Ariidae)	X		X		X	
*Corydoras paleatus *(Callichthyidae)	X					
*Horabagrus brachysoma *(*incertae sedis*)	X		X	X	X	
*Microglanis iheringi *(Pseudopimelodidae)	X			X		
*Noturus gyrinus *(Ictaluridae)	X	X		X	X	
*Pangasius hypophthalmus *(Pangasiidae)		X			X	
*Pimelodus pictus *(Pimelodidae)	X			X		
*Plotosus lineatus *(Plotosidae)			X			X
*Synodontis multipunctata *(Mochokidae)	X	X		X		

X denotes that the effect was observed in bass injected with 2 μL/g body weight of crude venom extract. In no case except that of *Plotosus lineatus *did injection of caudal fin extract produce any of the symptoms indicated above. In this species, injection of fin extract caused color loss, tetanus, loss of equilibrium, and eventual mortality.

The evolution of venom glands within the order Siluriformes was examined by performing maximum parsimony character optimization analyses on several previously published siluriform phylogenies that were reconstructed from both morphological [26,30] and molecular [28] data. Multiple phylogenies were analyzed due to the fact that the relationships of some siluriform families are either poorly resolved or vary between reported phylogenies. Given the widespread presence of venom glands in catfishes, it was expected that these previous systematic studies, in conjunction with the results presented above, would offer some insight into broader phylogenetic patterns of siluriform venom gland evolution in spite of the poor resolution of familial relationships found in these phylogenies.

**Figure 4:**
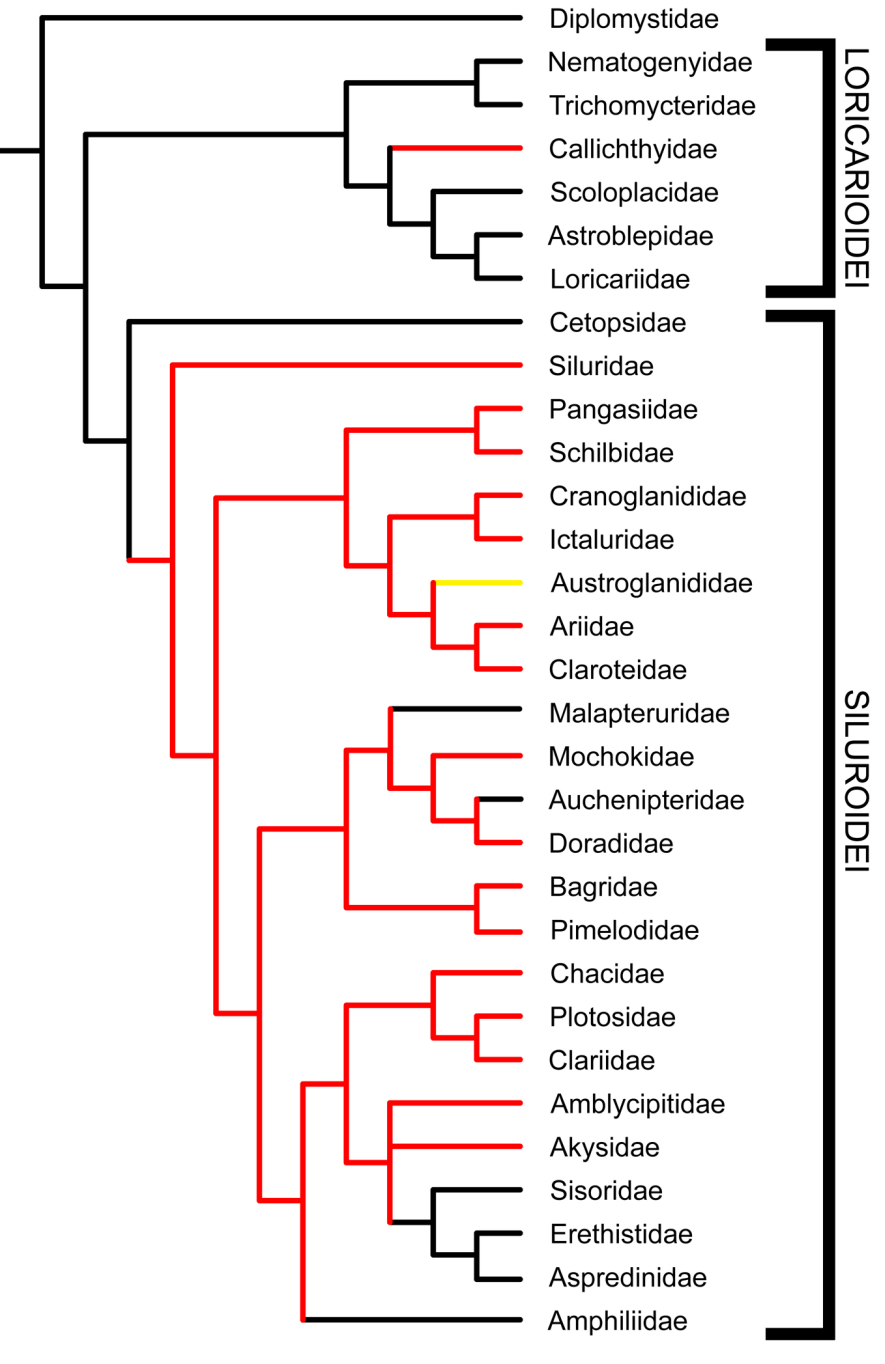
**Venom glands have evolved multiple times in catfishes**. The results of a character optimization analysis of a siluriform phylogeny generated from 440 morphological characters indicate the independent evolution of venom glands within the Loricarioidei as well as within the Siluroidei, leading to the majority of venomous catfish diversity. Phylogeny redrawn from Diogo [26]. Red branches indicate venomous lineages, black branches indicate non venomous lineages, yellow branches indicate lineages not examined in this study.

**Figure 5:**
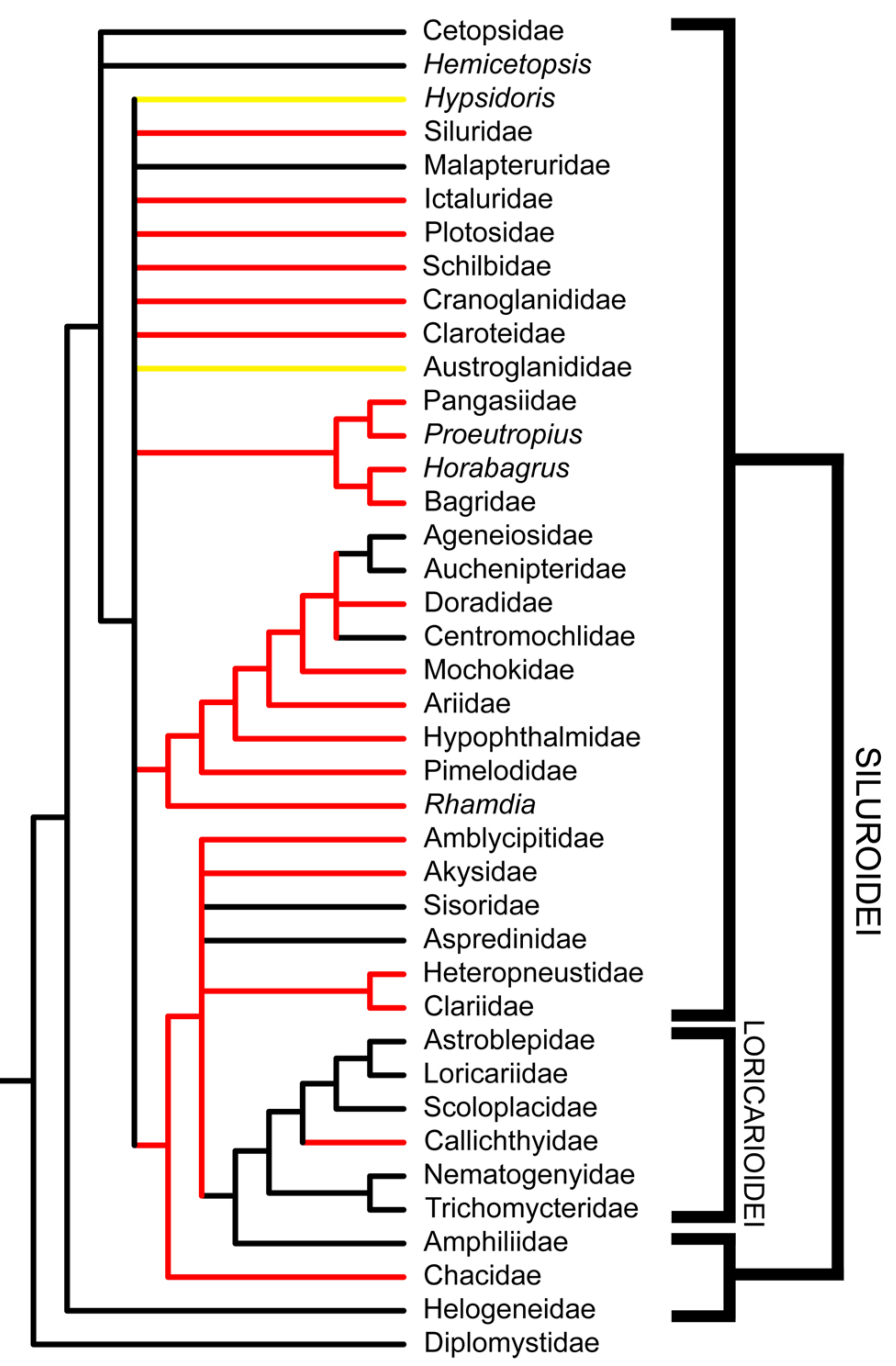
**Results of character optimization analysis using an alternative morphology-based phylogeny**. Phylogeny redrawn from Mo [30], based on 126 morphological characters. Red branches indicate venomous lineages, black branches indicate non venomous lineages, and yellow branches indicate groups not examined in this study. As in Figs. 4 and 6, the independent evolution of venom glands is indicated in the Loricarioidei (*sensu *[26] and [28]), in the family Callichthyidae. Patterns of venom gland evolution in the Siluroidei are obscured, due to the poor resolution of basal relationships. Given the broad range of siluroid families in which venom glands are found and similarities in venom composition between these families, a single, relatively basal development of venom glands seems the most parsimonious and likely scenario.

**Figure 6:**
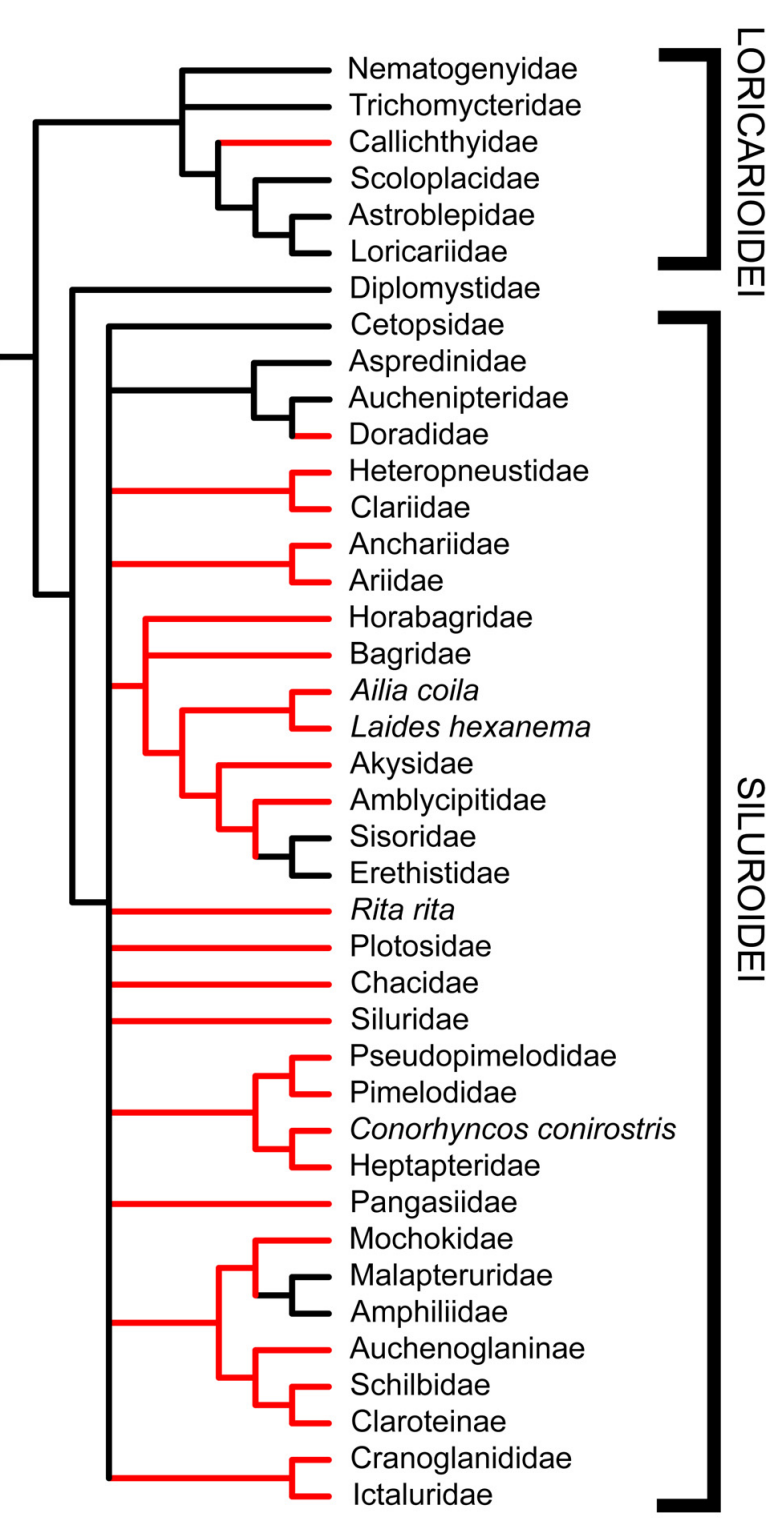
**Results of character optimization analysis using a recent molecular siluriform phylogeny**. Phylogeny redrawn from Sullivan et al. [28], based on RAG 1 and RAG 2 nuclear data. Red branches indicate venomous lineages, black branches indicate non venomous lineages. Again, the independent evolution of venom glands is found in the Loricarioidei, in the family Callichthyidae. Independent evolution of venom glands must also be ascribed to the family Doradidae, due to its nesting within a clade containing the non-venomous Aspredinidae and Auchenipteridae. Similarly to Fig. 5, the evolution of venom glands at the base of the Siluroidei are obscured, due to poor resolution of basal relationships.

Character optimization anlyses of these phylogenies indicate that this trait has arisen at least twice (Figs. 4, 5) and potentially three or more times (Fig. 6). Venom glands evolved once within the Loricarioidei, a diverse and exclusively Neotropical suborder of armored catfishes, in the family Callichthyidae. They also appear independently at least once basally within the Siluroidei, a clade containing all other non-loricarioid catfishes with the exception of the Diplomystidae. A recent molecular phylogeny based on nuclear gene sequences (RAG1 and RAG2) implies an additional evolution of venom glands within the Doradidae, owing to their placement within a clade of South American catfishes including the Aspredinidae and Auchenipteridae (members of which appear to lack venom glands) [[28]; Fig. 6].

**Figure 7:**
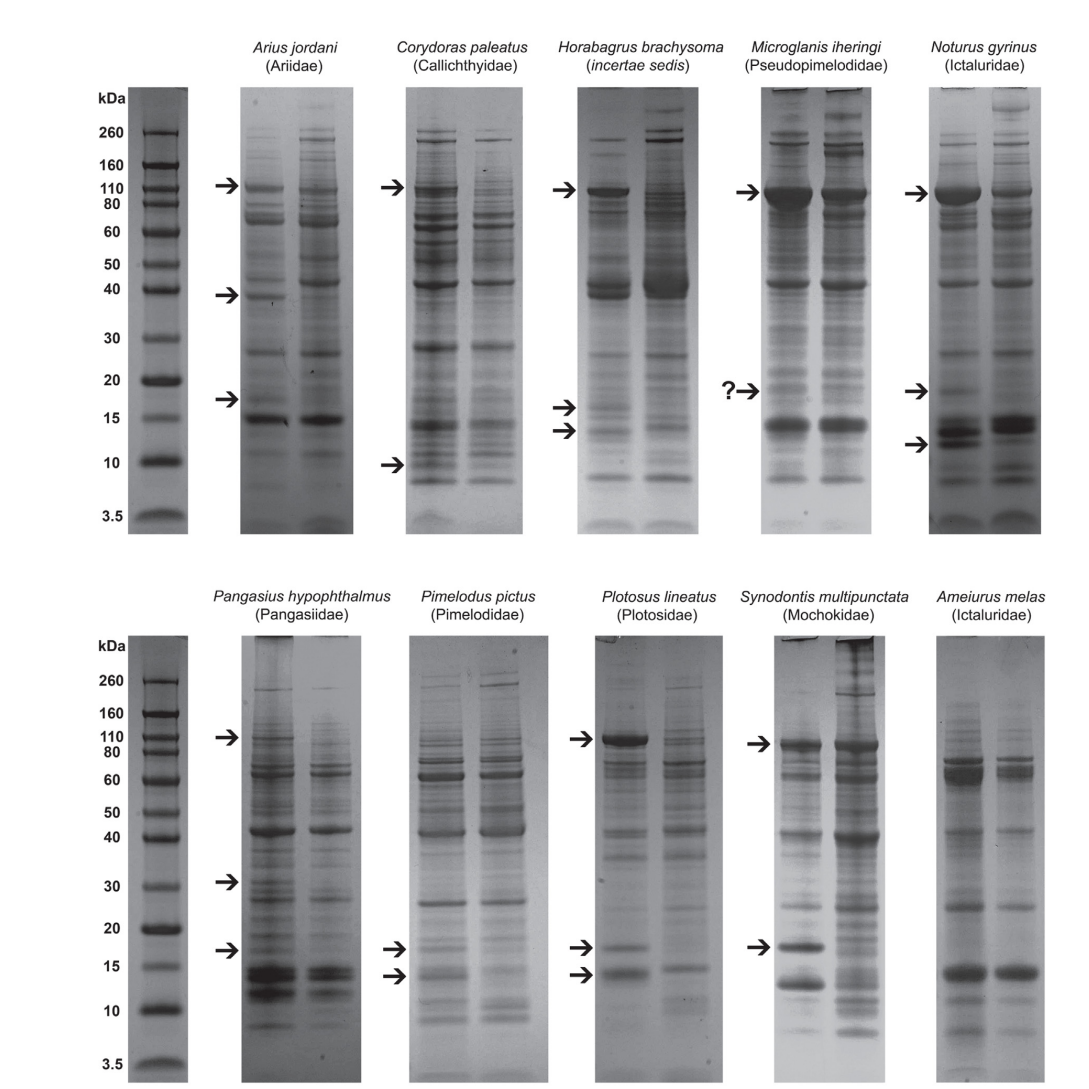
**SDS-PAGE analyses of venom extracts from several catfish species**. Left lanes represent venom extracts, right lanes represent extracts prepared from fin tissue. Arrows indicate positions of unique venom protein bands or proteins found in greater concentrations in venom extracts than in fin tissue extracts. (**?**) represents ambiguity between smearing and an additional, unique venom peptide band. Large quantities of a 110 kDa peptide are found in the venom extracts of nearly all species shown, with the exception of *Pimelodus*. The presence and variation of venom peptides in the size range of 10-20 kDa is also clearly visible. Samples from non-venomous *Ameiurus melas *are shown for comparison.

Sodium dodecyl sulfate polyacrylamide gel electrophoresis (SDS-PAGE) was used to identify venom proteins with similar molecular weights that are shared between species (and families), potentially reflecting homology of these proteins. Comparisons with extracts prepared from caudal-fin tissue were used to identify putative toxin peptides. The composition of different species' venoms was found to vary considerably, but some strong similarities were also evident. A putative toxin peptide of approximately 110 kDa was found in very high concentrations in the venom extracts of eight of the nine species examined (Fig. 7). Although a protein with a similar molecular weight was also found in the caudal-fin extracts of several species, it was generally found in much lower concentrations, and previous authors have stated that at least some toxin producing cells may be present in the fin tissue of catfishes [13]. In addition to the siluroid species tested, a 110 kDa peptide also appears to be present in the venom extracts of several species of *Corydoras*. *Corydoras *is distantly related to the remaining species analyzed, and the possession of venom glands by members of the family Callichthyidae appears to represent an independent evolution of these structures. A protein having this molecular weight was not found in the fin-spine extracts of *Pimelodus pictus*, a species shown by the current study to be venomous, reflecting a secondary loss of this putative toxic peptide. Additionally, nearly every siluroid species examined displayed at least one (and often more) putative toxic peptide(s) of approximately 10-20 kDa in weight. These peptides appear to vary significantly within this range however, and no molecular weight was represented with the same frequency as the 110 kDa peptide described above.

## Discussion

### Venomous Catfish Diversity

Examinations of histological sections of pectoral-fin spines, in conjunction with character optimization analyses of previously published siluriform phylogenies and toxicological assays, imply that approximately 1250-1625 species of catfishes from at least 20 families are venomous. These numbers are much higher than previous estimates, based largely on anecdotal evidence, which suggested a maximum of 1000+ venomous catfish species [18]. Of these families, 14 (Akysidae, Anchariidae, Callichthyidae, Chacidae, Claroteidae, Cranoglanididae, Doradidae, Heptapteridae, Mochokidae, Pangasiidae, Pimelodidae, Pseudopimelodidae, Schilbidae, Siluridae) are shown to contain venomous taxa for the first time; six (Amblycipitidae, Ariidae, Bagridae, Clariidae, Ictaluridae, Plotosidae) have previously been demonstrated to contain venomous representatives [20]. The approximation of 1250 species of venomous catfishes is undoubtedly an underestimate, as many genera in siluriform families containing venomous taxa remain to be examined. New species of catfishes are also continuously being discovered and described (958 species described in the last 10 years according to the Catalog of Fishes [31]), with some venomous genera such as *Chiloglanis *(Mochokidae) containing an estimated 25 or more undescribed species [J.P. Friel, pers. comm.].

**Figure 8:**
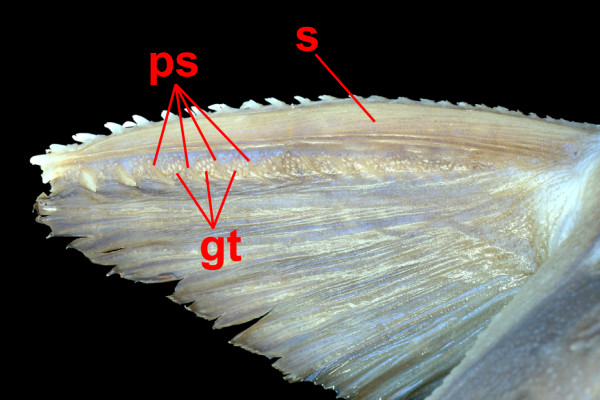
**The distinctive venom delivery apparatus of a doradid catfish**. Rather than forming longitudinal bundles along the spine, as in other siluroid catfishes, the glandular tissue in doradids is found in macroscopically visible aggregations between the posterior serrae of the fin spine. Abbreviations: s = pectoral spine, ps = posterior serrae, gt = glandular tissue.

The apparently low incidence of independent venom gland evolution in catfishes stands in stark contrast to the results obtained for venomous spiny-rayed fishes, in which venom glands appear to have evolved independently no fewer than nine times [18]. The exact number of times that venom glands arose within the Siluroidei remains ambiguous, though the majority of possible resolved topologies would require only a single derivation. However, the hypothesis of an additional derivation of venom glands in the family Doradidae that would be necessitated by the results of recent molecular phylogenetic analyses [28,29] does warrant further investigation. The venom glands found in doradid species differ morphologically from those seen in other siluroid families, by virtue of their structure (discrete clusters of glandular tissue internally subdivided into pockets of glandular cells by integumentary septa vs. continuous single sheaths of glandular cells) (Figs. 2 and 3), orientation (limited to spaces between posterior serrae of dorsal and pectoral-fin spines vs. being found along the entire length of the spines) (Fig. 8), and visibility without magnification (Fig. 8). Future studies of doradid venom composition should help to clarify this issue.

The loss of venom glands appears to be a common phenomenon within catfishes, which is not surprising given that bony fin spines have been lost in some families (Malapteruridae, most amphiliids). Genera in several families that contain venomous representatives (Heptapteridae, Pimelodidae, Siluridae) have also lost bony dorsal and/or pectoral-fin spines. Without an effective delivery system, there would seem to be no selection pressure for the maintenance of venom producing structures, leading to their reduction and eventual loss. The apparent loss of venom glands in groups that have maintained bony fin spines [Aspredinidae, Auchenipteridae, Sisoridae, some ictalurids (see Additional file 1)] is more unexpected, and explanations for these losses are not immediately apparent.

Inter- and intrageneric loss of venom glands was also found within the family Ictaluridae (Additional file 1). Both *Ameiurus melas *and *Pylodictus olivaris *lack any structures that could be identified as venom glands based on histological examination. Additionally, SDS-PAGE analysis detected no putative venom peptides in either species (Fig. 7). This finding was particularly surprising for *A. melas*, which had previously been considered venomous and quite virulent, based upon toxicological and histological work [13,20]. This discrepancy may be attributable to geographic variation in venom production; *A. melas *is a widely distributed species and the specimens examined in the current study were collected in Michigan, while those used in the previous toxicological study came from Texas. A potentially important factor in the case of *Pylodictus *is that this species can reach adult sizes that would presumably prohibit predation by even the largest North American predatory fishes (all of which are gape-limited predators), possibly weakening or eliminating selection for the maintenance of venom glands through adulthood.

The number of venomous catfishes estimated by this study (when combined with estimates of venomous spiny-rayed and cartilaginous fishes) supports previous claims that venomous fishes far outnumber all other venomous vertebrates [18], and also demonstrates that venomous catfish diversity likely equals or exceeds that of all other venomous vertebrates (including other fishes) combined (Table 3). Recently, some lizards and snakes traditionally considered to be non-venomous have been shown to produce several of the same toxic compounds as their venomous relatives [32]. Many of these species appear to lack a specialized mechanism for transmitting these compounds, possibly preventing them from being classified as venomous in the traditional sense [33], due to a potential inability to effectively utilize these compounds in feeding. However, recent work has shown that venom is likely to play a previously unsuspected, but major role in the feeding ecology of *Varanus komodoensis *(Komodo Dragon) [34]. This finding strongly indicates that such a role will be found for venom in other groups of lizards as well, potentially vastly increasing the estimate of venomous reptile diversity.

**Table 3:** Taxonomic distributions and estimates of venomous vertebrate diversity.

Taxon	# Presumed Venomous
Actinopterygii - Ray-finned fishes	≈1835 - 2275 species
Siluriformes - Catfishes	≈1250-1625 species
Acanthomorpha - Spiny-rayed fishes	≈585-650 species
Chondrichthyes - Cartilaginous fishes	≈200 species
Sarcopterygii - Lobe-finned fishes and tetrapods	≈685+ species

Estimates for acanthomorphs, chondrichthyans, and mammals are from [18]. Estimates for venomous snakes and lizards are from [[32] and [34]].

### Evolution of Catfish Venoms

Cameron and Endean [35] hypothesized that the venom glands of fishes are derived from glandular epidermal cells that secrete toxic proteinaceous compounds (termed "ichthyocrinotoxins") when fishes are threatened or injured. While it is true that these compounds are secreted in these situations, the hypothesis that they serve in an antipredatory capacity in catfishes appears flawed. With the exception of ichthyocrinotoxins associated with the epidermis of the dorsal and pectoral fin, there is no effective delivery device for these compounds, which are produced all over the body. This is of particular importance, as all assays demonstrating toxicity of epidermal secretions of catfishes have relied on intravenous injection of these compounds as a toxicological assay [36-40]. Furthermore, the presence of epidermal secretions does not appear to be a significant deterrent to potential predators, as they will attack and feed on distressed catfishes, as well as other baits coated with catfish epidermal secretions [[41]; pers. obs.].

That venom glands in catfishes produce similar compounds to epidermal glandular cells has been indicated by immunocytochemical assays [39]. The results of SDS-PAGE analyses presented here offer additional support for the similarity of these secretions. The major toxic factor of the skin secretion of *Arius bilineatus *has been isolated and shown to have a molecular weight of approximately 39 kDa [40]. The venom of *Arius jordani *clearly shows a strong band at approximately 39 kDa which is found in low concentration in the control lane (Fig. 7). The presence of this protein in the control sample is likely due to the presence of epidermal secretory cells in the tissue sample used, while the low concentration is due to the removal of most of the epidermal secretions before sample preparation. While these cells were also probably present in spine samples, the large difference in concentration indicates that venom gland cells are likely responsible for production of most of this protein band. A similar case is seen in the electrophoretic profile of *Plotosus lineatus*, which shows major toxin bands at 15-16 kDa and 13-14 kDa (Fig. 7). While the larger band is similar in weight to toxin-PC, as characterized by Auddy and Gomes [22], the lower band is very similar in weight to a toxic fraction isolated from the skin secretions of this species [37,38], with the slight discrepancy in estimated size possibly being due to differences in sample preparation and analysis.

While the venom gland cells in catfishes (and other fishes as well) are likely to be derived from epidermal secretory cells, an alternative scenario to Cameron and Endean's antipredatory hypothesis is also able to explain their origin. Studies of the epidermal secretions of several *Arius *species have indicated that these compounds are able to accelerate healing of wounds and may also have some antimicrobial properties [41-43]. The spines of catfishes act to effectively increase their cross-sectional circumference when locked into place, and would likely be the first structures to contact a gape-limited predator's tissues during an attack. As such, the spines would often be damaged, and individuals with larger numbers of epidermal secretory cells surrounding the spine could gain a selective advantage due to decreased healing time and a corresponding decreased chance of infection of exposed tissues. This selection may have led to increased aggregations of these cells around the fin spines, with the toxic effects of their secretions being an epiphenomenon to their primary healing benefits. Once the toxic secretions had become associated with an effective delivery device, selection for increased toxicity, as seen in some plotosid and clariid species, could begin to operate. Explicit tests of this scenario will require more detailed structural and genetic characterizations of these compounds.

The symptoms of envenomation produced by a diverse array of catfish species' venoms are very similar and a large number of putative toxins appear to fall within a well-defined molecular weight range. The conserved molecular weight patterns and toxic effects of catfish venom peptides suggest two possible scenarios for the evolution of venoms in catfishes: widespread convergent evolution of catfish venom toxins with similar targets and thus similar molecular characteristics and effects, or common origins of toxic peptides with subsequent species-specific alterations. The widespread presence of venom glands shown by the character optimization evidence discussed above strongly suggests that the latter case is the more parsimonious and likely scenario, even in cases where phylogenetic resolution of basal siluriform divergences is lacking.

## Conclusion

This study utilizes several lines of investigation to increase our knowledge of several poorly understood areas of the biology of venomous catfishes. These investigations have demonstrated that at least 1250, and possibly over 1600 species of catfishes may be venomous, a number far greater than any previous estimate of venomous catfish diversity. In conjunction with previous systematic studies, these findings also offer insight into the evolutionary history of venom glands in the order Siluriformes, indicating at least two independent evolutionary origins of these structures. Finally, the symptoms of catfish envenomation, along with preliminary biochemical characterizations of toxic catfish venom peptides, may suggest a novel selective explanation for the evolution of catfish venom glands and their secretions.

Finer-scale studies of venom gland evolution in fishes will require continued systematic studies of venomous fish families to elucidate the relationships of the species contained therein. Additionally, examinations of the chemical composition of fish venoms and the identities and structures of their constituents will provide valuable insight into the mechanisms and potential selective factors driving venom evolution in fishes, as well as their potential for biomedical research and pharmaceutical bioprospecting.

## Methods

### Venom Gland Survey and Histological Techniques

The right pectoral-fin spine was removed from 158 catfish specimens (see Additional file 1), housed in the fish collection of the University of Michigan Museum of Zoology. Spines were decalcified in CalEx^® ^according to the manufacturer's instructions, after which segments from the distal third of the spine of an appropriate size for histological preparation were removed. These segments were subjected to automated dehydration and paraffin infiltration and embedding at the Tissue Core Facility of the University of Michigan Comprehensive Cancer Center. Serial sections of 0.7 microns were then obtained from each spine sample. Sections were stained with hematoxylin and eosin and mounted on glass slides.

Spines were examined for the presence of venom glands using a Nikon YS2-T compound microscope. Morphological confirmation of the presence of venom gland cells was achieved by comparisons with previously published photomicrographs of venom glands in catfishes and spiny rayed fishes [20,35,44,45], descriptions of piscine venom gland cellular anatomy [20], and sections obtained from the spines of catfish species that have been shown to secrete venomous substances by previous studies [13,20]. When a representative of a particular genus was found to possess venom glands, all members of that genus were presumed to be venomous, except in the case of the ictalurid genus *Ameiurus*, where the examination of multiple species within the genus indicated otherwise. These generic counts of venomous species formed the basis for the minimum estimate of venomous catfish species (Table 1). The number of species contained in unexamined genera from families containing venomous representatives was added to the minimum estimate to give a maximum estimate of venomous catfish species (Table 1).

### Venom gland extract preparation and assay

Representatives of the catfish families Ariidae, Bagridae, Callichthyidae, Ictaluridae, Mochokidae, Pangasiidae, Pimelodidae, Pseudopimelodidae, and Plotosidae were obtained either from field collections (Ictaluridae) or the aquarium trade (other families). Specimens were euthanized using MS-222 at a concentration of 300 mg/L in fresh water. All further preparations were carried out either on ice or under refrigeration at 4°C. Spines and caudal fin tissue were removed from each specimen, rinsed in physiological saline and gently scraped with a microspatula in order to remove any excess epidermal secretions, and weighed to the nearest 0.001 g using a GeneMate digital balance. Spines were minced and then further homogenized in a 2 mL Dounce homogenizer along with either marine (Plotosidae) or freshwater (other families) euteleost physiological saline at a volume of 2 mL/g of tissue [46]. The homogenate was then centrifuged at 6,000 rpm at 4°C for 20 minutes and the supernatant collected. The supernatant served as the crude venom extract. Control extracts prepared from caudal fin tissue were prepared in the same manner.

Largemouth Bass were collected from Boyden Creek, Washtenaw Co., MI in October of 2008. Bass were anesthetized in MS-222 at a concentration of 75 mg/L of fresh water and weighed to the nearest 0.1 g. They were then placed in 10 G experimental aquaria in a room with natural light and allowed to acclimate for a period of 72 hours. After the 72 hour acclimation period, bass were injected in the caudal peduncle at a depth of 2 mm with 2 μL/g body weight of either crude venom extract or control extract. Individuals were then observed at one minute, one hour, and 24 hours after injection for symptoms consistent with envenomation (Table 2). For each species of catfish tested, two bass were injected with venom extract and two were injected with caudal fin control extract.

### Character Optimization Analyses

Several previously published phylogenetic hypotheses for the order Siluriformes [26,28,30] were examined using MacClade 4.0 PPC [47]. Presence and absence of venom glands was traced onto the trees using the criterion of maximum parsimony. Specific taxa that were present in the phylogenetic reconstruction but which were not examined in the current study were coded as ambiguous (?) within the data matrix.

### SDS-PAGE Analyses

Crude extracts were prepared for SDS-PAGE analysis by reduction with NuPAGE^® ^reducing agent and loading buffer, according to manufacturer's instructions. Reduced samples were subjected to electophoresis in NuPAGE^® ^precast 4-12% Bis-Tris polyacrylamide gels in 1× MES running buffer for 35 minutes, at 200 V in an x-Cell SureLock™ Mini Cell. Reduced peptides were visualized using SimplyBlue™ SafeStain according to manufacturer's instructions. Molecular weights of venom and caudal fin extracts were estimated by comparison with Novex^® ^Sharp Protein Standard. Proteins unique to venom extracts (relative to caudal-fin extracts) were treated as putative toxins, pending further characterization.

## Supplementary Material

Additional file 1 **List of catfish specimens from which histological preparations were made and examined**. Presence or absence of a bony spine capable of effectively delivering venomous secretions is noted, as is a brief description of the condition of venom glands, in each specimen found to possess them. Taxonomic assignments follow [19]. Click here for file
