# Supplementary material for: Diversity, phylogenetic distribution, and origins of venomous catfishes
Source: BMC Evol Biol. 2009 Dec 4;9:282. doi: 10.1186/1471-2148-9-282 (PMC2791775; doi:10.1186/1471-2148-9-282)
Supplement: Additional file 1 — List of catfish specimens from which histological preparations were made and examined. Presence or absence of a bony spine capable of effectively delivering venomous secretions is noted, as is a brief description of the condition of venom glands, in each specimen found to possess them. Taxonomic assignments follow [19]. [file 1471-2148-9-282-S1.DOC]

**Table S1**

| ***Taxon*** | ***Bony Spine*** | ***Venom Gland Condition*** | ***Museum Voucher*** |
| --- | --- | --- | --- |
| Akysidae |  |  |  |
| *Acrochordonichthys rugosus* | Y | Paired, in anterior grooves | UMMZ 245670 |
| *Akysis hendricksoni* | Y | Paired, in anterior grooves | UMMZ 238793 |
| *Breitensteinia cessator* | Y | Spine complete, but lacking glandular cells and anterior grooves | UMMZ 243238 |
| *Parakysis anomalopteryx* | Y | Glands stripped, well-developed anterior grooves present | UMMZ 209923 |
| *Pseudobagarius inermis* | Y | Paired, in anterior grooves | UMMZ 234709 |
| *Pseudobagarius similis* | Y | Paired, in anterior grooves | UMMZ 241324 |
| Amblycipitidae |  |  |  |
| *Amblyceps mangois* | Y | Paired, in anterior grooves | UMMZ 244760 |
| *Liobagrus mediadiposalis* | Y | Paired, in anterior grooves | UMMZ 238983 |
| *Liobagrus reini* | Y | Paired, in anterior grooves | UMMZ 183862 |
| Amphiliidae |  |  |  |
| *Amphilius uranoscopus* | N | No discernible glandular cells | UMMZ 199996 |
| *Leptoglanis rotundiceps* | Y | No discernible glandular cells | UMMZ 200020 |
| Anchariidae |  |  |  |
| *Gogo ornatus* | Y | Multiple bundles of glandular cells along posterior half of spine | UMMZ 244995 |
|  |  |  |  |
|  |  |  |  |
|  |  |  |  |
| ***Taxon*** | ***Bony Spine*** | ***Venom Gland Condition*** | ***Museum Voucher*** |
| Ariidae |  |  |  |
| *Arius aguadulce* | Y | Paired anterior glands | UMMZ 143460 |
| *Bagre marinus* | Y | Paired anterior glands | UMMZ 244720 |
| *Batrachocephalus mino* | Y | Spine damaged, anterior glandular cell remnants visible | UMMZ 155787 |
| *Cochlefelis danielsi* | Y | Spine damaged, posterior glandular cells visible | UMMZ 214019 |
| *Hemipimelodus borneensis* | Y | Small, paired, posterior glands | UMMZ 214617 |
| *Osteogeniosus militaris* | Y | Spine damaged, posterior glandular cells visible | UMMZ 245436 |
| *Potamarius nelsoni* | Y | Spine damaged, anterior glandular cells visible | UMMZ 143498 |
|  |  |  |  |
| Aspredinidae |  |  |  |
| *Bunocephalus rugosus* | Y | No discernible glandular cells | UMMZ 206289 |
| *Dysichthys bifidus* | Y | No discernible glandular cells | UMMZ 204374 |
| Astroblepidae |  |  |  |
| *Astroblepus chotae* | N | No discernible glandular cells | UMMZ 179260 |
| Auchenipteridae |  |  |  |
| *Ageneiosus sp.* | Y | No discernible glandular cells | UMMZ 240342 |
| *Centromochlus sp.* | Y | No discernible glandular cells | UMMZ 214828 |
| *Entomocorus benjamini* | Y | No discernible glandular cells | UMMZ 204709 |
| *Parauchenipterus striatulus* | Y | No discernible glandular cells | UMMZ 216166 |
| *Trachelyopterus coriaceus* | Y | No discernible glandular cells | UMMZ 216161 |
| ***Taxon*** | ***Bony Spine*** | ***Venom Gland Condition*** | ***Museum Voucher*** |
| Bagridae |  |  |  |
| *Bagrichthys majusculus* | Y | Paired anterior and posterior glands | UMMZ 241720 |
| *Bagrus docmac* | Y | Anterior glandular cells present | UMMZ 187332 |
| *Batasio affinis* | Y | Small, paired, posterior glands | UMMZ 245967 |
| *Hemibagrus spilopterus* | Y | Paired anterior and posterior glands | UMMZ 238651 |
| *Hyalobagrus flavus* | Y | Spine damaged, posterior glandular cells visible | UMMZ 248500 |
| *Mystus mysticetus* | Y | Spine damaged, anterior and posterior glandular cells visible | UMMZ 232730 |
| *Nanobagrus nebulosus* | Y | Single circumferential gland | UMMZ 238794 |
| *Olyra longicaudata* | Y | Glandular cells near anterior groove | UMMZ 243657 |
| *Pseudomystus siamensis* | Y | Paired hemispherical glands | UMMZ 224812 |
| *Rama chandramara* | Y | Single circumferential gland | UMMZ 247463 |
| *Rita rita* | Y | Spine damaged, large, paired, posterior glands visible | UMMZ 244943 |
| *Sperata aor* | Y | Spine stripped, no glandular cells visible | UMMZ 208359 |
| *Tachysurus crassilabris* | Y | Paired anterior and posterior glands | UMMZ 232107 |
| *Tachysurus intermedius* | Y | Paired anterior and posterior glands | UMMZ 245073 |
|  |  |  |  |
|  |  |  |  |
| ***Taxon*** | ***Bony Spine*** | ***Venom Gland Condition*** | ***Museum Voucher*** |
| Callichthyidae |  |  |  |
| *Aspidoras taurus* | Y | Circumferential glandular cells | UMMZ 236693 |
| *Callichthys callichthys* | Y | Circumferential glandular cells | UMMZ 235769 |
| *Corydoras aeneus* | Y | Circumferential glandular cells | UMMZ 205959 |
| *Corydoras splendens* | Y | Spine stripped, no glandular cells visible | UMMZ 235764 |
| *Dianema longibarbis* | Y | Paired posterior glands, a few anterior glandular cells | UMMZ 235768 |
| *Hoplosternum littorale* | Y | Unorganized anterior glandular cells | UMMZ 207376 |
| Cetopsidae |  |  |  |
| *Cetopsis plumbea* | N | No discernible glandular cells | UMMZ 203882 |
| *Helogenes marmoratus* | N | No discernible glandular cells | UMMZ 232086 |
| Chacidae |  |  |  |
| *Chaca chaca* | Y | Paired anterior glands | UMMZ 244665 |
| Clariidae |  |  |  |
| *Clarias theodorae* | Y | Paired hemispherical glands | UMMZ 200160 |
| *Dinotopterus cunningtoni* | Y | Circumferential glandular cells | UMMZ 199927 |
| *Encheloclarias velatus* | Y | Spine stripped, no glandular cells visible | UMMZ 243883 |
| *Gymnallabes typus* | Y | Circumferential glandular cells | UMMZ 243235 |
| *Heterobranchus longifilis* | Y | Spine broken, glandular cells present | UMMZ 189155 |
| ***Taxon*** | ***Bony Spine*** | ***Venom Gland Condition*** | ***Museum Voucher*** |
| Clariidae (cont.) |  |  |  |
| *Heteropneustes fossilis* | Y | Small, paired, posterior glands | UMMZ 209199 |
| *Tanganikallabes sp.* | Y | Single large, circumferential gland | UMMZ 196021 |
| *Xenoclarias holobranchus* | Y | Spine damaged, ventral hemispherical gland visible | UMMZ 187331 |
| Claroteidae |  |  |  |
| *Auchenoglanis occidentalis* | Y | Paired posterior glands, small anterior gland also visible | UMMZ 200182 |
| *Bathybagrus tetranema* | Y | Spine damaged, posterior glands visible | UMMZ 196092 |
| *Chrysichthys mabusi* | Y | Spine damaged, posterior glands visible | UMMZ 200184 |
| *Clarotes laticeps* | Y | Paired, anterior glands visible, posterior portion of spine stripped | UMMZ 195024 |
| *Lophiobagrus cyclurus* | Y | Spine damaged, large, hemispherical glands evident | UMMZ 199932 |
| Cranoglanididae |  |  |  |
| *Cranoglanis henrici* | Y | Spine damaged, lateral gland remnants visible | UMMZ 238763 |
| Diplomystidae |  |  |  |
| *Diplomystes nahuelbutaensis* | Y | No discernible glandular cells | UMMZ 227170 |
| ***Taxon*** | ***Bony Spine*** | ***Venom Gland Condition*** | ***Museum Voucher*** |
| Doradidae |  |  |  |
| *Amblydoras hancockii* | Y | Glandular tissue between posterior serrae | UMMZ 66314 |
| *Doras micropoeus* | Y | Glandular tissue between posterior serrae | UMMZ 216217 |
| *Leptodoras nelsoni* | Y | Glandular tissue between posterior serrae | UMMZ 245737 |
| *Lithodoras dorsalis* | Y | Glandular tissue between posterior serrae | UMMZ 230776 |
| *Opsodoras humeralis* | Y | Glandular tissue between posterior serrae | UMMZ 216894 |
| *Physopyxis lyra* | Y | Spine stripped, no glandular cells visible | UMMZ 204520 |
| *Trachydoras paraguayensis* | Y | Glandular tissue between posterior serrae | UMMZ 207842 |
| Heptapteridae |  |  |  |
| *Imparfinis lineata* | N | Posterior glandular cells visible | UMMZ 194201 |
| *Myoglanis sp.* | Y | Single large, circumferential gland | UMMZ 231759 |
| *Pimelodella mucosa* | Y | Spine damaged, paired, large, circumferential glands visible | UMMZ 207033 |
| *Rhamdia guatemalensis* | Y | Paired anterior and posterior glands | UMMZ 193940 |
|  |  |  |  |
|  |  |  |  |
| ***Taxon*** | ***Bony Spine*** | ***Venom Gland Condition*** | ***Museum Voucher*** |
| Ictaluridae |  |  |  |
| *Ameiurus brunneus* | Y | No discernible glandular cells | UMMZ 156102 |
| *Ameiurus* *melas* | Y | No discernible glandular cells | UMMZ 243064 |
| *Ameiurus* *natalis* | Y | Paired, in anterior grooves | UMMZ 233519 |
| *Ameiurus nebulosus* | Y | Paired, in anterior grooves | UMMZ 230890 |
| *Ameiurus platycephalus* | Y | Paired, in anterior grooves | UMMZ 225986 |
| *Ameiurus serracanthus* | Y | 4 distinct pairs, surrounding spine | UMMZ 186261 |
| *Ictalurus furcatus* | Y | Paired, in anterior grooves | UMMZ 201496 |
| *Ictalurus pricei* | Y | Spine damaged anteriorly, anterior groove visible, lacking glandular cells, glandular tissue visible posteriorly |  |
| *Ictalurus punctatus* | Y | Paired, in anterior grooves | UMMZ 114940 |
| *Noturus albater* | Y | Paired, in anterior grooves | UMMZ 167170 |
| *Noturus elegans* | Y | Paired, in anterior grooves | UMMZ 165395 |
| *Noturus eleutherus* | Y | Spine damaged anteriorly, well-developed anterior groove visible, lacking glandular cells, glandular tissue visible posteriorly | UMMZ 66624 |
| *Noturus flavus* | Y | Paired, in poorly-developed anterior groove | UMMZ 199203 |
| *Noturus furiosus* | Y | Large hemispherical glands | UMMZ 107097 |
| *Noturus insignis* | Y | Paired, in anterior grooves | UMMZ 36151 |
| *Noturus leptacanthus* | Y | Paired, in anterior grooves | UMMZ 242770 |
| ***Taxon*** | ***Bony Spine*** | ***Venom Gland Condition*** | ***Museum Voucher*** |
| Ictaluridae (cont.) |  |  |  |
| *Noturus munitus* | Y | Paired, in anterior grooves | UMMZ 181771 |
| *Noturus nocturnus* | Y | Paired, in anterior grooves | UMMZ 242696 |
| *Noturus placidus* | Y | Paired, in anterior grooves | UMMZ 167656 |
| *Noturus stigmosus* | Y | Paired, in multiple grooves | UMMZ 248603 |
| *Pylodictis olivaris* | Y | Spine damaged, no glands evident | UMMZ 226324 |
| Loricariidae |  |  |  |
| *Ancistrus cirrhosus* | Y | No discernible glandular cells | UMMZ 204398 |
| *Aphanotorulus unicolor* | Y | No discernible glandular cells | UMMZ 205129 |
| *Farlowella kneri* | Y | No discernible glandular cells | UMMZ 206541 |
| *Hemipsilichthys sp.* | Y | No discernible glandular cells | UMMZ 215265 |
| *Hypostomus boulengeri* | Y | No discernible glandular cells | UMMZ 207649 |
| *Loricaria cataphracta* | Y | Possible glandular cells in posterior groove | UMMZ 207475 |
| *Otocinclus vittatus* | Y | No discernible glandular cells | UMMZ 216577 |
| Malapteruridae |  |  |  |
| *Malapterurus tanganyikaensis* | N | No discernible glandular cells | UMMZ 199858 |
|  |  |  |  |
| Mochokidae |  |  |  |
| *Chiloglanis productus* | Y | Paired anterior and posterior glands | UMMZ 199817 |
| *Euchilichthys astatodon* | Y | No discernible glandular cells | UMMZ 195064 |
| *Microsynodontis batesii* | Y | Spine stripped, no glands visible | UMMZ 248519 |
| ***Taxon*** | ***Bony Spine*** | ***Venom Gland Condition*** | ***Museum Voucher*** |
| Mochokidae (cont.) |  |  |  |
| *Mochokiella paynei* | Y | Spine stripped, no glands visible | UMMZ 248513 |
| *Synodontis irsacae* | Y | Paired anterior and posterior glands | UMMZ 199829 |
| *Synodontis* *zambesensis* | Y | Paired anterior glands | UMMZ 200003 |
|  |  |  |  |
| Nematogenyidae |  |  |  |
| *Nematogenys inermis* | N | A few possibly glandular cells | UMMZ 212697 |
| Pangasiidae |  |  |  |
| *Helicophagus leptorhynchus* | Y | Spine damaged, paired, hemispherical glands evident | UMMZ 214467 |
| *Pangasianodon hypophthalmus* | Y | Paired, hemispherical glands | UMMZ 232681 |
| *Pangasius bocourti* | Y | Paired, hemispherical glands | UMMZ 234583 |
| *Pseudolais pleurotaenia* | Y | Paired, hemispherical glands | UMMZ 214260 |
| Pimelodidae |  |  |  |
| *Cheirocerus eques* | Y | Spine stripped, no glands visible | UMMZ 187223 |
| *Hypopthalmus marginatus* | Y | Spine stripped, no glands visible | UMMZ 231726 |
| *Megalonema platinum* | N | No discernible glandular cells | UMMZ 216638 |
| *Parapimelodus valencienesi* | Y | Spine stripped, no glands visible | UMMZ 218468 |
| *Pimelodus clarias* | Y | Paired anterior and posterior glands | UMMZ 211343 |
| *Sorubim lima* | Y | Paired anterior and posterior glands | UMMZ 242595 |
|  |  |  |  |
| ***Taxon*** | ***Bony Spine*** | ***Venom Gland Condition*** | ***Museum Voucher*** |
| Plotosidae |  |  |  |
| *Paraplotosus albilabris* | Y | Large, paired, dorsal and ventral glands | UMMZ 100219 |
| *Plotosus canius* | Y | Large, paired, hemispherical glands | UMMZ 245508 |
| Pseudopimelodidae |  |  |  |
| *Pseudopimelodus zungaro* | Y | Paired, small, anterior glands | UMMZ 206076 |
| Schilbidae |  |  |  |
| *Ailia coilia* | Y | Spine stripped, no glands visible | UMMZ 244694 |
| *Clupisoma garua* | Y | Spine stripped, no glands visible | UMMZ 208292 |
| *Eutropiichthys vacha* | Y | Spine stripped, no glands visible | UMMZ 208330 |
| *Laides longibarbis* | Y | Spine damaged, small, paired, posterior glands visible | UMMZ 235391 |
| *Neotropius atherinoides* | Y | Small, paired, posterior glands | UMMZ 208591 |
| *Pseudeutropius brachypopterus* | Y | Spine damaged, paired posterior glands visible | UMMZ 243440 |
| *Schilbe mystus* | Y | Spine damaged, large hemispherical gland visible | UMMZ 200312 |
| *Silonia silondia* | Y | Spine damaged, glandular cells visible | UMMZ 208460 |
| *Siluranodon auritus* | Y | Spine stripped, no glands visible | UMMZ 195044 |
| Scoloplacidae |  |  |  |
| *Scoloplax empousa* | Y | No discernible glandular cells | UMMZ 214696 |
| ***Taxon*** | ***Bony Spine*** | ***Venom Gland Condition*** | ***Museum Voucher*** |
| Siluridae |  |  |  |
| *Belodontichthys truncatus* | N | No discernible glandular cells | UMMZ 217151 |
| *Hito taytayensis* | Y | Large, posterior gland | UMMZ 100557 |
| *Kryptopterus bicirrhis* | Y | Spine damaged, posterior glandular cells visible | UMMZ 241757 |
| *Ompok bimaculatus* | Y | Spine damaged, posterior glands visible | UMMZ 240771 |
| *Ompok krattensis* | Y | Spine damaged, posterior glands visible | UMMZ 238655 |
| *Pterocryptis berdmorei* | Y | Paired, large, posterior glands | UMMZ 246494 |
| *Pterocryptis cochinchinensis* | Y | Paired, large, posterior glands | UMMZ 248529 |
| *Silurichthys schneideri* | Y | Paired, large, posterior glands | UMMZ 243747 |
| *Silurus asotus* | Y | Spine damaged, paired anterior glands visible, posterior glandular cells also visible | UMMZ 180202 |
| *Silurus mento* | Y | Spine stripped, no glands visible | UMMZ 214491 |
| *Wallago micropogon* | N | No discernible glandular cells | UMMZ 186807 |
| Sisoridae |  |  |  |
| *Ayarnangra estuarius* | Y | No discernible glandular cells | UMMZ 248520 |
| *Bagarius yarelli* | Y | No discernible glandular cells | UMMZ 241095 |
| *Caelatoglanis zonatus* | Y | No discernible glandular cells | UMMZ 247116 |
| *Conta conta* | Y | No discernible glandular cells | UMMZ 247195 |
| *Erethistes pusillus* | Y | No discernible glandular cells | UMMZ 247198 |
| *Gagata sexualis* | Y | No discernible glandular cells | UMMZ 244895 |
| *Glyptothorax panda* | Y | No discernible glandular cells | UMMZ 246004 |
| ***Taxon*** | ***Bony Spine*** | ***Venom Gland Condition*** | ***Museum Voucher*** |
| Sisoridae (cont.) |  |  |  |
| *Glyptothorax platypogonides* | Y | No discernible glandular cells | UMMZ 235704 |
| *Gogangra viridescens* | Y | No discernible glandular cells | UMMZ 243717 |
| *Hara hara* | Y | No discernible glandular cells | UMMZ 247446 |
| *Sisor rabdophorus* | Y | No discernible glandular cells | UMMZ 240013 |
| Trichomycteridae |  |  |  |
| *Trichomycterus areolatus* | N | No discernible glandular cells | UMMZ 215412 |
|  |  |  |  |
| *Incertae sedis* |  |  |  |
| *Horabagrus brachysoma* | Y | Paired anterior and posterior glands | UMMZ 247478 |
